# Quantitative Estimation of Protein in Sprouts of *Vigna radiate* (Mung Beans), *Lens culinaris* (Lentils), and *Cicer arietinum* (Chickpeas) by Kjeldahl and Lowry Methods

**DOI:** 10.3390/molecules27030814

**Published:** 2022-01-26

**Authors:** Nayab Batool Rizvi, Samina Aleem, Mohammad Rizwan Khan, Sadia Ashraf, Rosa Busquets

**Affiliations:** 1 School of Chemistry, New Campus, University of the Punjab, Lahore 54590, Pakistan; samchaudhry295@outlook.com; 2 Department of Chemistry, College of Science, King Saud University, P.O. Box 2455, Riyadh 11451, Saudi Arabia; 3 Department of Earth and Environmental Science, Bahria University, Islamabad 44000, Pakistan; sadiaashraf.pgc@gmail.com; 4 School of Life Sciences, Pharmacy and Chemistry, Kingston University London, Penrhyn Road, Kingston Upon Thames, London KTI 2EE, UK; r.busquets@kingston.ac.uk

**Keywords:** protein, mung beans, lentils, chickpeas, Kjeldahl method, Lowry method

## Abstract

Protein scarcity is the most vital cause of long-lasting diseases and even untimely deaths in some developing nations. The application of protein in food is advantageous from the point of view of non-toxicity, biocompatibility, and dietary benefits. This study aimed to determine the protein contents of the sprouts of *Vigna radiates* (mung beans), *Lens culinaris* (lentils), and *Cicer arietinum* (chickpeas) using the Kjeldahl and Lowry methods. The results obtained from the Kjeldahl method identified protein concentrations of 2.54, 2.63, and 2.19%, whereas the Lowry method results identified protein concentrations of 2.96%, 4.10%, and 1.6% in mung beans, lentils, and chickpeas, respectively. In both the methods, lentils were found to have the highest amount of protein followed by mung beans and chickpeas. Both the Kjeldahl and Lowry methods demonstrated good protein values and low variation in the protein amount in the analyzed samples. Furthermore, the methods had greater sensitivity and comparable experimental variability. The outcomes revealed that assays can be applied for protein analysis in legumes. In the context of a lack of suitable standard procedures for evaluating legumes’ compositions, the present study is suitable for food control laboratories. In addition, the studied samples represent a significant source of protein and can be used to fulfil the daily requirements for protein intake and other food applications.

## 1. Introduction

Every cell of the human body contains protein, which acts like tiny machines within each cell [1]. The human body’s skin, muscles (in the form of actin and myosin), bones (in the form of collagen), blood stream (in the form of hemoglobin and plasma albumin, etc.), and other parts contain a significant amount of protein [1,2]. Protein accounts for almost 20% of the total body weight [3,4]. Enzymes, polypeptide hormones, and immunoglobulins (antibodies) are also made up of proteins [5]. Protein is also found in the form of neurotransmitters and oxygen carriers in the blood stream [6,7]. Protein deficiency is a major cause of illness and premature death in some developing countries [7,8]. It leads to mental retardation and a reduced IQ level [7,8,9].

Mung beans, lentils, and chickpeas belong to the legume family, and can be eaten in various ways, such as fermented, cooked, and milled into flour [10]. Germination also increases the legumes’ nutritive value by promoting the formation of enzymes, which eradicate or decrease the antinutritional and indigestible issues in legumes [11,12,13]. Dehulling, soaking, germination, cooking, microwave cooking, and autoclaving change the chemical composition, antinutritional factors, and dietary feature of mung beans [13]. However, dehulling and soaking practices result in less minerals being lost than cooking methods [14,15,16,17]. Mung beans, lentils, and chickpeas have greater antioxidant activities and alimentary values compared to other legumes. They have the potential to inhibit many chronic illnesses including cardiovascular disease, obesity, and cancer [11,18,19,20]. Legumes are an enriched source of protein [16,21,22,23,24,25]. Generally, mature legumes contain more protein [23], and the constituent amino acids determine the quality of the protein [26,27,28]. A complete protein provides the body with all essential amino acids (EAAs) [29,30,31,32]. Plant source protein contains less specific EAAs [33,34,35,36] and protein digestion from plant sources is also comparatively lower than animal protein [37,38,39,40]. Legume protein is also low in sulfur-containing amino acids, for instance, methionine, tryptophan, and cysteine [41,42,43]. Proteins from the legumes can be improved nutritionally through different processes, for instance, in the form of flours and baked goods [42,44,45]. The objective of the current study was to determine the protein concentration in sprouts of *Vigna radiates* (mung beans), *Lens culinaris* (lentils), and *Cicer arietinum* (chickpeas) using the Kjeldahl and Lowry methods. The results revealed that the assays can be applied for protein analysis in legumes, particularly mung beans, lentils, and chickpeas, and are highly suitable for food control research laboratories and to assess the quality of legumes.

## 2. Results

### 2.1. Kjeldahl Method

**Figure 1:**
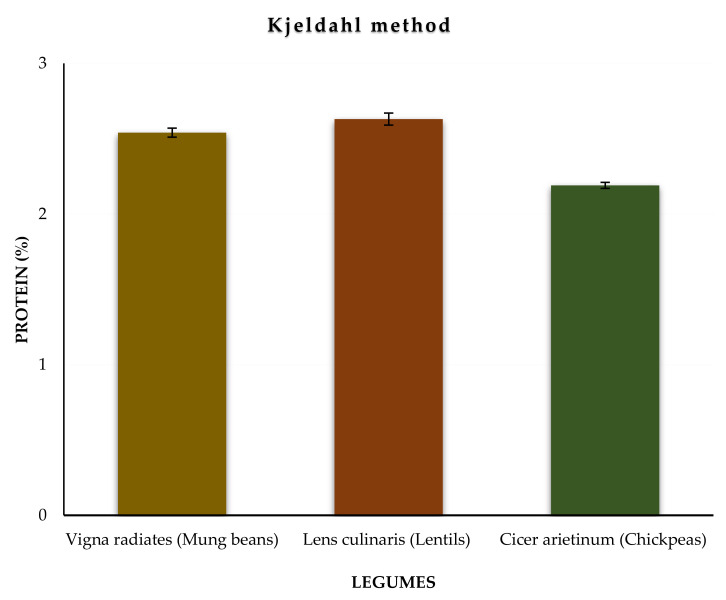
Variation of the protein level in the studied samples using the Kjeldahl method.

Protein (%) from mung beans, lentils, and chickpeas was calculated using the formulae described in Section 3.4.1. In 2 g samples of mung beans, lentils, and chickpeas, the protein (%) amounts were found to be 2.54, 2.63, and 2.19%, respectively. The variation of the protein level in the studied samples is illustrated in Figure 1. The outcomes revealed that mung beans and lentils contain nearly the same amount of protein, whereas chickpeas contain a relatively lower amount of protein. The results are presented in Table 1.

### 2.2. Lowry Method

**Figure 2:**
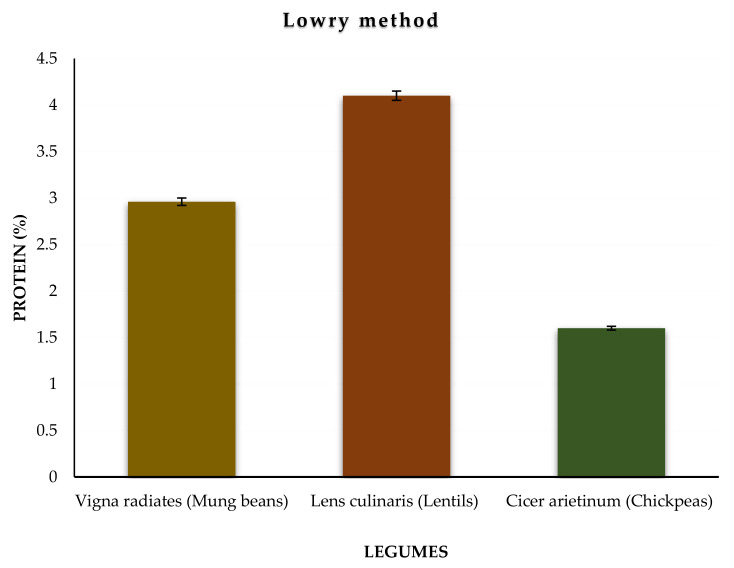
Variation of the protein level in the studied samples using the Lowry method.

The absorbance of the calibration solutions (0.2–1.0 µg/mL) and samples was measured at the wavelength (λmax) of 660 nm using a spectrophotometer. The λmax of the calibration solutions was found to be 0.074–0.275 nm, whereas the λmax values of the studied samples of mung beans, lentils, and chickpeas were 0.159, 0.221, and 0.110 nm, respectively. The standards and samples were analyzed in six replicates (*n* = 6). The amounts of protein in mung beans (2.96%), lentils (4.10%), and chickpeas (1.60%) were obtained. The variation of the protein level in the studied samples is illustrated in Figure 2. Relatively, mung beans and lentils produce higher amounts of protein than chickpeas (Table 2).

### 2.3. Experimental Statistical Analysis

Statistical analysis of the samples was performed. The standard deviation of mung beans, lentils, and chickpeas was found to be 0.08, 0.08, and 0.08, respectively. The obtained method variables of the studied samples are demonstrated in Table 3. The correlation values (paired samples correlation) among mung beans, lentils, and chickpeas are presented in Table 4. The significance values at the 95% confidence levels among these pairs are illustrated in Table 5. The system resulted in significant method variables for the studied samples.

## 3. Materials and Methods

### 3.1. Chemicals and Reagents

All reagents used were of analytical and/or reagent grade. Sulfuric acid (H_2_SO_4_, >999%), sodium hydroxide (NaOH, ≥98%, pellets), and hydrochloric acid (HCl) were obtained from Sigma-Aldrich (Darmstadt, Germany). Potassium sulfate (K_2_SO_4_), copper sulfate (CuSO_4_), sodium carbonate, and methyl red were purchased from BHD Chemicals (Poole, England). Bovine serum albumin (BSA) and egg albumin were purchased from a local supermarket (Lahore, Pakistan). An analytical balance model TX323L was obtained from Shimadzu (Tokyo, Japan). A spectrophotometer was obtained from Metash (Shanghai, China).

### 3.2. Protein Standard 

Protein standard stock solution (1000 µg/mL) was prepared using bovine serum albumin/egg albumin 0.1 g (dry weight), dissolved in distilled water, and diluted with distilled water (100 mL). The sample dilution was carried out for calibration purposes. The method linearity was studied between 0.2 µg/mL and 1.0 µg/mL. Concentrations were converted to percent values and applied accordingly.

### 3.3. Sample Collection and Preparation

Mung beans (*Vigna radiate*, also known as *Phaseolusaureus* L.), lentils (*Lens culinaris*, also known as ‘Mash beans’), and black chickpeas (*Cicerarietinum*) are popular food legumes in Asian countries. Samples were obtained from Toba Tek Singh, Punjab Pakistan. Impurities were removed from the samples followed by disinfection with detergent and distilled water.

#### Sample Soaking and Sprouting

Sanitized grains were soaked in distilled water (1:3 *w*/*v*) for 24 h. Subsequently, the water was drained out and the grains were placed in sterilized petri dishes that contained moist filter paper and left to sprout in a culture room at 25 °C ± 2 for a period of 6–12 days (approximately 144–300 h). Adequate moisture is necessary for the sprouting of grains, which was provided by the moist filter papers. After 6 days, mung bean sprouts (approximately 2–3 inches in length) were collected and finely ground by a mortar piston. The resulting materials were used for further chemical analysis. Mash beans and black grams were collected after 12 days (approximately 2 inches in length), ground by a mortar piston, and used for further chemical analysis.

### 3.4. Chemical Analysis

#### 3.4.1. Kjeldahl Method

Sample digestion was carried out by assessing nearly 2 g of sample in a digestion flask with sulfuric acid (12–15 mL) followed by the addition of 7 g of potassium sulfate and copper sulfate. The sample was heated at 370 °C to 400 °C using a heating block. Once white fumes started to appear, then the samples were heated for a further 60–90 min. After, the sample was cooled by cautiously adding 250 mL of water.

Distillation was performed by adding an appropriate volume of precisely measured acid standard solution (15 mL) to water (70 mL) followed by the addition of methyl red indicator (3 to 4 drops) to the solution. Then, sodium hydroxide (80 mL) solution was added to the digested mixture to make it strongly alkali. The flask was immediately connected to a distillation apparatus and distilled until 150 mL of distillate were collected in the titrating flask. Then, the digestion and titrating flasks were removed from the unit, rinsing the condenser tube with distilled water.

As the ammonia dissolved in the acid trapping solution, it neutralized some of the HCl. The remaining solution was titrated with standard, a known solution of base (NaOH). The amount of ammonia distilled from the digestive solution was measured followed by the determination of the nitrogen level in the protein.

Samples were digested using a strong acid so that it released nitrogen, which can be determined by a suitable titration technique. The amount of protein was calculated from the nitrogen concentration of the food. Briefly, 1 mole of ammonia impending from the digestion mixture was neutralized exactly with 1 mole of the acid in the trapping flask. The first calculation, therefore, aimed to identify the number of moles of ammonia that were produced and then trapped from the sample(s). To estimate the initial number of moles of acid in the trapping flask (before any ammonia was trapped), the molarity of the acid solution was multiplied by the volume of the trapping solution. To calculate the number of moles of base (NaOH) that were added from the burette to neutralize the remaining acid (that not neutralized by the ammonia), the “moles of base” added from the “moles of acid” present at the beginning were subtracted. To obtain the moles of ammonia, the number of “moles of ammonia” from the sample was the same as the “moles of nitrogen”. Then, the number of grams of nitrogen in the original sample of protein was calculated by multiplying the “moles of nitrogen” by the atomic mass of nitrogen (mass of atoms of nitrogen). The following formulae were applied to calculate the nitrogen mass, percentage, and amounts of crude protein:

Mass of nitrogen = (moles of ammonia) (moles of N/moles of NH_3_) (14.01 g N/moles of N) (1)

Nitrogen (%) = Mass of N in sample/Mass of analyzed sample × 100(2)

The amount of “crude protein” (CP) was found by multiplying the percent nitrogen by a factor F (usually 6.25):

Crude protein (CP%) = % N × F(3)

where F = 6.25 for all forages and feeds and 5.70 for wheat grains.

#### 3.4.2. Protein Extraction

Protein extraction from the legumes was carried by a subsequent process. Primarily, the samples were cleaned followed by grinding and sieving. After, a known amount of the sieved sample was mixed with Milli-Q water (5 mL), and the pH of the sample solution was regulated to be pH 9 using sodium hydroxide (NaOH, 0.1 N) at 25 °C. Then, the solution was shaken for 50 min at room temperature followed by centrifugation (HERMLE, model Z32 HK, Wehingen, Germany) at 10,000 rpm for 10 min. The sample extraction and centrifugation methods were repeated twice for the residue to achieve better yields. In order to precipitate the protein, sample extracts were collected together and the pH value (4.5) was adjusted using hydrochloric acid HCl (1 N). Proteins was obtained by eradication of the sample supernatant by means of decantation. The achieved protein mass was cleaned two times with Milli-Q water and further centrifuged (10,000 rpm, 15 min). The obtained protein was then freeze-dried using a freeze-dryer model BenchTop Pro with Omnitronics (SP Scientific, New York, NY, USA), and used for further analysis [46,47].

Lowry’s reagent A: (2% Na_2_CO_3_ in 0.1 N NaOH) was prepared by the addition of NaOH (2 g) and Na_2_CO_3_ (10 g) in distilled water (5 mL) followed by dilution to 500 mL with distilled water. Lowry’s reagent B_1_ was prepared with the addition of CuSO_4_ (1 g) and distilled water (5 mL), diluted to 100 mL with distilled water. Lowry’s reagent B_2_ was prepared with the addition of sodium potassium tartrate (2 mL) and distilled water (5 mL), diluted to 100 mL with distilled water. Lowry’s reagent C was freshly prepared with the addition of Lowry’s reagent B_1_ (2 mL) and Lowry’s reagent B_2_ (2 mL) while stirring solution was added to Lowry’s reagent A (200 mL). Then, 2 g of the extracted protein sample were added to reagent C followed by incubation for 45 min with continuous stirring in a dark room at room temperature. Then, reagent E (1 mL) was added to the sample, and incubated again for 45 min. Finally, the amounts of protein in the samples were determined using a spectrophotometer.

This method was performed by measuring the absorbance of all standards and samples using a spectrophotometer. The phenolic group of tyrosine and tryptophan residues (amino acid) in a protein produced a blue purple color complex, which had a maximum absorption in the region of the 660 nm wavelength with Folin–Ciocalteau reagent, which consists of sodium tungstate molybdate and phosphate. Thus, the intensity of the color depends on the amount of these aromatic amino acids present and will thus vary for different proteins. The reaction is dependent on pH and a working range of pH 9 to 10.5 is essential.

## 4. Discussion

Relating to the antinutritional factors in grains, harmful substances exist in the grain, which affect biomolecules’ absorption and obstruct their bioavailability to humans [48]. Legumes comprise antinutritional factors, for instance, lectins, protease inhibitors, oxalate, total free phenolics, tannins, cyanogens, antivitamins, phytic acid, toxic amino acids, and saponins [49]. These chemicals decrease the availability and digestibility of protein and cause antinutritional issues. Nevertheless, in legumes, a small number of antinutritional factors have been described to have health advantages. Consequently, these secondary metabolites are currently recognized as functional food constituents [49].

Mung beans contain many essential amino acids, but antinutritional factors limit its application. To overcome these problems, dehulling of the seeds is performed before milling [50]. Legumes have low nutritive values because they have antinutritional factors and less sulfur-containing amino acids and protein digestibility [51]. Cooking is conducted to improve the quality of protein by destroying the structures of antinutritional factors [52]. However, cooking also causes a loss of soluble nutritional factors, such as minerals and vitamins [53]. An increase in the temperature and time also results in a loss of nutritional factors and essential amino acids from the proteins of legumes [54].

Germination enhances the nutritional values of legumes. It induces the formation of enzymes that eliminate the antinutritional factors in legumes [12]. Dehulling, cooking, germination, autoclaving, and microwave cooking affect the composition, quality, and nutritional and antinutritional factors of mung beans. The loss of minerals in the dehulling and soaking process is reduced compared to the cooking process. Therefore, the recommended processes for mung beans are autoclaving and microwave cooking. These processes not only improve the quality of mung beans but also their cooking time [13]. According to our study, using the Kjeldahl method, the mung bean sample contained 2.54% crude protein. The Lowry method obtained a 2.96% sample concentration. The amount of protein determined in mung beans is in accordance with past literature [55,56]. Mung beans are known for their significant health benefits, containing approximately 20–25% protein, and albumin and globulin represent the main protein stores. In addition, mung bean protein comprises a higher amount of essential amino acids, for instance, methionine, tryptophan, lysine, phenylalanine, arginine leucine, isoleucine, and valine. Therefore, mung bean consumption has significantly increased in recent years [55].

Chickpeas are the most economic (21.7–23.4%) protein source [57]. As such, researchers have shown greater interest in chickpeas [58]. Chickpea seeds contain many antinutritional factors, such as protease, lectins, amylase inhibitors, and polyphenols. Phytic acids and certain sugars, such as stachyose and raffinose, are also antinutritional factors found in chickpea seeds [58]. They inhibit the essential amino acids from reacting, resulting in fewer applications of chickpea seeds in different food products [58]. To overcome this problem, chickpea proteins can be isolated, and chickpeas proteins can form a gel in higher concentrations using convective drying. Denaturation of protein can improve EAI and ESI. This is why CPCs can be used for nutraceutical and functional food applications [59]. The Kjeldahl method identified 2.19% crude protein and the Lowry method identified approximately 1.6% protein. Relatively, the amount of protein found in chickpeas was less than the protein amount found in mung beans. These values were in good agreement with values reported in a previous study [57].

The use of protein in food applications is beneficial from the point of view of biocompatibility, non-toxicity, and nutritional advantages. Due to the amphoteric property of protein, folate (vitamin B9) was encapsulated. To obtain microencapsulates, the homogenous mixture of folate and protein was acidified to the isoelectric point. The encapsulation efficiency and loading capacity were calculated to be 62.19 ± 2.05% and 10.18 ± 0.89%, respectively. Encapsulation enhances the stability of folate [60].

Lentils have high protein contents with remarkable functional properties. Germination results in changes in the composition and nutritional values of lentils. This increases the activity of phytase but decreases the phytic acid activity. Extractable minerals include iron, calcium, magnesium, and phosphorus [61]. Albumin is the major protein in lentils followed by globulin. Cooking decreases the albumin content accompanied by a significant increment in the glutelin fractions. SDS-PAGE of cooked lentil protein fractions showed that lentil protein was qualitatively and quantitatively altered after cooking. The total number of subunit proteins before cooking was 17 to 19 bands and after cooking, it ranged from 13 to 16 bands [62].

Using the Kjeldahl method, the amount of protein determined in the lentils sample was 2.63%. This amount was found to be higher than the values obtained in mung beans and chickpeas. However, the Lowry method results showed a higher amount of protein of 4.10%. Among the analyzed samples, lentils contained a higher amount of protein than chickpeas. The applied methods (Kjeldahl and Lowry) demonstrated greater values of protein and the lowest variation in the protein amount in the analyzed samples. Furthermore, both the methods had greater sensitivity and comparable variability. The outcomes revealed that assays can be applied for routine protein analysis in legumes.

## 5. Conclusions

From the results, it is concluded that the studied legumes (mung beans, lentils, and chickpeas) represent an enriched source of protein. Both applied methods (Kjeldahl and Lowry) were correlated, and offered almost similar outcomes. The Kjeldahl method showed that the concentrations of protein were 2.54, 2.63, and 2.19% in mung beans, lentils, and chickpeas, whereas the Lowry method found protein concentrations of 2.96%, 4.10%, and 1.6% in mung beans, lentils, and chickpeas, respectively. In both methods, lentils were found to have the highest amount of protein followed by mung beans and chickpeas. Both the Kjeldahl and Lowry methods showed significant protein levels and low variation of the protein contained in the analyzed samples. In addition, the methods showed greater sensitivity and comparable experimental variability. The results revealed that assays can be applied for protein analysis in legumes, particularly mung beans, lentils, and chickpeas. Thus, they can be used to fulfil daily requirements for protein intake and other food applications.

## Figures and Tables

**Table 1:** Amount of protein in mung beans, lentils, and chickpeas using the Kjeldahl method.

Method	Sample	Protein (%) ± SD	Mean Value (Protein, %) ± SD
Kjeldahl	Mung beans	2.54 ± 0.03	2.45 ± 0.03
	Lentils	2.63 ± 0.04	
	Chickpeas	2.19 ± 0.02	

SD, standard deviation (*n* = 6).

**Table 2:** Amount of protein in mung beans, lentils, and chickpeas using the Lowry method.

Method	Sample	Protein (%) ± SD	Mean Value (Protein, %) ± SD
Lowry	Mung beans	2.96 ± 0.04	2.88 ± 0.03
	Lentils	4.10 ± 0.05	
	Chickpeas	1.60 ± 0.02	

SD, standard deviation (*n* = 6).

**Table 3:** Method variables of the analyzed samples (mung beans, lentils, and chickpeas).

Method Variables	Mung	Lentils	Chickpeas
N	6	6	6
Mean *	0.15	0.16	0.14
Median	0.16	0.16	0.13
Standard deviation	0.08	0.08	0.08
Variance	0.01	0.01	0.01
Skewness	0.23	0.12	0.56
Standard error of skewness	0.80	0.80	0.80
Range	0.00	0.23	0.23

N = number of samples; * (%).

**Table 4:** Paired sample correlation values among mung beans, lentils, and chickpeas.

Sample Pair	N	Correlation	Sig.
Pair 1, mung and lentils	6	0.96	0.002
Pair 2, mung and chickpeas	6	0.97	0.001
Pair 3, lentils and chickpeas	6	0.87	0.025

N = number of samples; Sig., significance.

**Table 5:** Significance values at the 95% confidence level among the paired samples.

	Paired Differences					t	df	Sig. (2-Tailed)
	Mean	Std. Dev.	Std. Error Mean	95% Confidence Interval of the Difference				
				Lower	Upper			
Pair 1, mung and lentils	0.10	0.03	0.10	0.37	0.02	1.00	5	0.36
Pair 2, mung and chickpeas	0.01	0.02	0.01	0.13	0.03	1.00	5	0.36
Pair 3, lentils and chickpeas	0.19	0.05	0.19	0.03	0.07	1.00	5	0.36

Std. Dev., standard deviation; t, t-value; df, degrees of freedom; Sig., significance.

## Data Availability

Not applicable.
